# Local Control by Mediastinal Lymph-Node Dissection in Stage I Non-Small-Cell Lung Cancer

**DOI:** 10.1093/icvts/ivag189

**Published:** 2026-07-21

**Authors:** Shunsuke Morita, Takahiro Mimae, Yujin Kudo, Takuya Nagashima, Norifumi Tsubokawa, Yoshihiro Miyata, Hiroyuki Ito, Norihiko Ikeda, Morihito Okada

**Affiliations:** Department of Surgical Oncology, Hiroshima University, Hiroshima, 734-8551, Japan; Department of Surgical Oncology, Hiroshima University, Hiroshima, 734-8551, Japan; Department of Surgery, Tokyo Medical University, Tokyo, 160-0023, Japan; Department of Thoracic Surgery, Kanagawa Cancer Center, Yokohama, 241-8515, Japan; Department of Surgical Oncology, Hiroshima University, Hiroshima, 734-8551, Japan; Department of Surgical Oncology, Hiroshima University, Hiroshima, 734-8551, Japan; Department of Thoracic Surgery, Kanagawa Cancer Center, Yokohama, 241-8515, Japan; Department of Surgery, Tokyo Medical University, Tokyo, 160-0023, Japan; Department of Surgical Oncology, Hiroshima University, Hiroshima, 734-8551, Japan

**Keywords:** lobectomy, locoregional recurrence, prognosis, mediastinal lymph-node dissection, non-small-cell lung cancer

## Abstract

**Objectives:**

Mediastinal lymph-node dissection (MLND) is recommended for resectable non-small-cell lung cancer (NSCLC); however, its prognostic impact remains controversial. We evaluated whether MLND improves prognosis in patients with radiologically solid-dominant clinical stage I NSCLC.

**Methods:**

We retrospectively reviewed 2212 patients who underwent lobectomy for solid-dominant clinical stage I NSCLC between 2010 and 2022. Prognosis and local control were compared between the MLND and non-MLND groups.

**Results:**

The MLND and non-MLND groups included 1912 and 300 patients, respectively. After propensity score matching, 5-year overall survival did not show a statistically significant difference between the groups (85.3% vs 79.3%, *P* = .089). MLND identified occult pN2 metastasis in 7.3% (138/1912) of patients. Notably, 3.6% (68/1912) of the entire MLND cohort had pN2 disease yet remained recurrence-free. Furthermore, the MLND group demonstrated significantly lower mediastinal lymph-node recurrence than the non-MLND group (*P* = .017). Among patients with pN2 disease in the MLND group, those with recurrence (*n* = 70) had a higher incidence of intrapulmonary metastases (*P* = .042), a higher number of mediastinal lymph-node metastases (*P* = .002), and a lower rate of skip-N2 involvement (*P* = .031).

**Conclusions:**

Mediastinal lymph-node dissection provides superior local control by reducing mediastinal recurrences and enables accurate pathological staging. While this advantage guides appropriate adjuvant therapy selection, the direct impact of MLND on overall survival remains uncertain. Its true survival benefit should be evaluated in future prospective randomized trials.

## Graphical abstract

**Figure:**
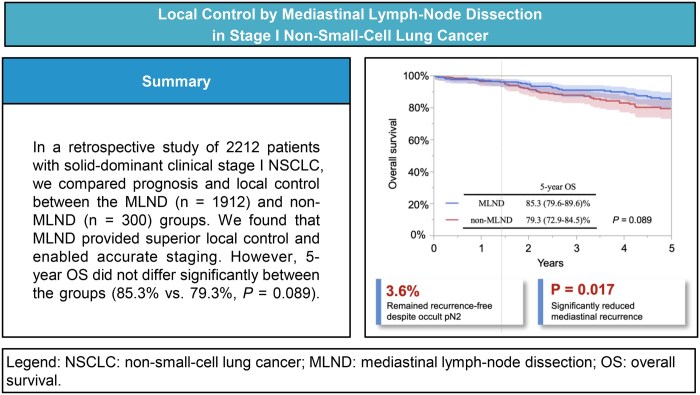

## INTRODUCTION

The standard treatment for patients with resectable non-small-cell lung cancer (NSCLC) is anatomical surgical resection, including lobectomy or segmentectomy with hilar and mediastinal lymph-node dissection (MLND) or sampling (MLNS).^1^ Although the importance of accurate staging through MLND has been demonstrated, its impact on prognosis remains controversial.^2–5^ Several meta-analyses comparing MLND with MLNS have reported inconsistent findings.^6–8^ Moreover, the ACOSOG Z0030 trial, a multicentre prospective randomized controlled trial, did not demonstrate a better prognosis among patients who underwent MLND than in those who underwent MLNS.^5^

In patients with clinically suspected lymph-node metastasis, MLND is essential for accurate staging, and adjuvant therapy becomes beneficial once nodal involvement is pathologically confirmed. Conversely, in patients with clinical N0, the risk of hilar or mediastinal metastasis is influenced by the consolidation tumour ratio (CTR). Ground-glass opacity (GGO)-dominant tumours (CTR ≤ 0.5) are associated with a low risk of nodal metastasis, and omission of MLND has been proposed for this subgroup.^9^^,^^10^ By contrast, solid-dominant tumours (CTR > 0.5) are associated with a higher risk of hilar or mediastinal lymph-node metastasis, and MLND may improve prognosis through enhanced local control.^11^

However, whether MLND contributes to improved prognosis in patients with radiologically solid-dominant clinical stage I NSCLC remains uncertain, as evidence specific to this subgroup is limited. We aimed to evaluate whether MLND confers a prognostic benefit by comparing outcomes between patients with radiologically solid-dominant clinical stage I NSCLC who underwent MLND and those who did not.

## METHODS

### Ethical statement

The Institutional Review Boards of the participating institutions approved this retrospective analysis of a prospective database and waived the requirement to obtain informed consent from individual patients (E2018-1216-02; November 30, 2022).

### Patient enrolment

In this multicentre retrospective study, we used data from the HITOKA-3 Project Database, which includes records from Kanagawa Cancer Center (Kanagawa, Japan), Tokyo Medical University (Tokyo, Japan), and Hiroshima University Hospital (Hiroshima, Japan).

Medical records of patients with radiologically solid-dominant clinical stage I NSCLC who underwent curative lobectomy between January 2010 and December 2022 were analysed. Preoperative staging was based on high-resolution CT (HRCT) and 18-fluoro-2-deoxyglucose positron emission tomography/CT (FDG-PET/CT). Tumours were staged according to the eighth edition of the TNM classification for malignant tumours.^12^^,^^13^ Lymph-node metastasis was considered negative when the short axis of the mediastinal or hilar lymph nodes measured <1 cm on HRCT and when no FDG uptake was observed in these lymph nodes on FDG-PET/CT. Bronchoscopy or mediastinoscopy was performed when the evaluation of lymph-node metastasis was challenging. We excluded patients who underwent preoperative treatment and sublobar resections, including wedge resection, segmentectomy, and incomplete resection (R1 or R2). Adjuvant therapy was administered to patients with a pathological tumour size >2 cm or pathological stages II-IIIB.

### Surgical procedure

Lobe-specific lymph-node dissection (ND2a-1) or systematic lymph-node dissection (ND2a-2) was classified as MLND, whereas MLNS was not. The decision to perform MLND depended on the surgeon’s discretion, based on a comprehensive evaluation of the patient’s background and tumour characteristics.

### Follow-up evaluation

Beginning the day after lung resection, all patients underwent quarterly follow-ups, with physical examinations and chest radiography, as well as chest and abdominal CT scans every 6 months for the first 2 years. Subsequent follow-up included biannual chest radiography, physical examinations, and an annual chest CT scan. Recurrence was classified as either locoregional or distant. Locoregional recurrence was defined as recurrence in the preserved lobe or ipsilateral hilar or mediastinal lymph nodes, whereas distant recurrence was defined as any recurrence outside these regions.

### Statistical analysis

Continuous variables were reported as medians (interquartile range [IQR]) and compared using the Mann-Whitney *U*-test. Categorical variables were reported as counts (percentages) and compared using Fisher’s exact test. Overall survival (OS) was defined as the time from surgery to death from any cause or censored at the last follow-up. Recurrence-free survival (RFS) was defined as the time from surgery to recurrence, death from any cause, or censored at the last follow-up. Survival data were estimated using the Kaplan-Meier method and compared using the log-rank test. In the multivariable proportional hazards model, clinical variables such as age, sex, pack-years, solid tumour size, maximum standardized uptake value (SUVmax), presence of GGO, cT stage, and MLND status were included in the analysis. The analysis was performed using the backward stepwise method. To adjust for baseline characteristics, the propensity score was estimated using a logistic regression model based on factors related to tumour malignancy, including age (continuous), sex (male or female), solid tumour size (continuous), presence of GGO (absent or present), and SUVmax (continuous). Propensity score matching (PSM) was performed in a 1:1 ratio, using greedy matching with a calliper width of 0.20 times the standard deviation of the logit-transformed estimated propensity score. Standardized differences were calculated to assess the balance between patient characteristics. All statistical analyses were performed using JMP Pro version 18.1 (SAS Institute, Cary, NC, United States) and EZR version 1.68 (Saitama Medical Center, Jichi Medical University), a graphical user interface for R (R Foundation for Statistical Computing, Vienna, Austria). Statistical significance was set at *P* < .05.

## RESULTS

### Patient characteristics

The characteristics of the 2212 patients are summarized in **Table 1**. Of these, 1912 and 300 were included in the MLND and non-MLND groups, respectively. Patients in the non-MLND group were significantly older than those in the MLND group (*P* < .001). No significant differences were observed between the 2 groups in terms of sex (*P* = .605), pack-years (*P* = .401), laterality (*P* = .477), presence of GGO (*P* = .103), and SUVmax (*P* = .221). The MLND group exhibited significantly larger whole tumour and solid tumour sizes than the non-MLND group (*P* = .028 and *P* = .005, respectively), as well as a more advanced cT stage (*P* = .006). Regarding the pathological backgrounds, no significant differences were observed between the groups in terms of histology (*P* = .260), whole tumour size (*P* = .336), pleural invasion (*P* = .103), or pulmonary metastasis (*P* = .477). However, the MLND group exhibited a higher frequency of lymphatic and vascular invasion (*P* = .005 and *P* = .018, respectively), more advanced pN stage (*P* < .001), and a higher rate of adjuvant therapy (*P* < .001) than the non-MLND group. After PSM, the MLND group demonstrated a more advanced pN stage (*P* < .001) and a higher rate of adjuvant therapy (*P* < .001) than the non-MLND group. No significant differences were observed in other variables. **Table 1** outlines the characteristics of the 282 patients in each group after PSM.

**Table 1.** Patient Characteristics in the MLND and Non-MLND Groups Before and After Propensity Score Matching

	Before propensity score matching				After propensity score matching		
	MLND (*n* = 1912)	Non-MLND (*n* = 300)	*P*-value	SMD	MLND (*n* = 282)	Non-MLND (*n* = 282)	*P*-value
Age, years, median (IQR)	69 (62-74)	77 (71-81)	<.001	0.060	76 (70-79)	76 (70-80)	.992
Sex, *n* (%)			.605	0.094			.735
Female	849 (44)	138 (46)			127 (45)	131 (46)	
Male	1063 (56)	162 (54)			155 (55)	151 (54)	
Pack-year, *n*, median (IQR)	22 (0-47)	30 (0-50)	.401	–	19 (0-51)	31 (0-50)	.333
Side, *n* (%)			.477	–			.161
Right	1241 (65)	201 (67)			172 (61)	188 (67)	
Left	671 (35)	99 (33)			110 (39)	94 (33)	
Clinical whole tumour size, cm, median (IQR)	2.4 (1.8-3.1)	2.3 (1.6-3.1)	.028	–	2.3 (1.8-3.0)	2.3 (1.7-3.1)	.576
Solid tumour size, cm, median (IQR)	2.2 (1.6-2.8)	1.98 (1.4-2.7)	.005	0.053	2.1 (1.5-2.7)	2.0 (1.4-2.8)	.852
GGO, *n* (%)			.103	0.138			1.000
Absent	1197 (63)	173 (58)			167 (59)	167 (59)	
Present	715 (37)	127 (42)			115 (41)	115 (41)	
SUVmax, median (IQR)	4.2 (2.1-8.1)	3.3 (1.7-7.6)	.221	0.164	3.3 (1.8-6.9)	3.4 (1.6-8.0)	.175
cT factor (eighth), *n* (%)			.006	–			.569
T1mi	1 (0.1)	1 (0.3)			0 (0.0)	1 (0.4)	
T1a	87 (4.6)	25 (8.3)			18 (6.4)	22 (7.8)	
T1b	757 (39.6)	135 (45.0)			117 (41.5)	122 (43.3)	
T1c	666 (34.8)	84 (24.0)			95 (33.7)	82 (29.1)	
T2a	401 (21.0)	55 (16.9)			52 (18.4)	55 (19.5)	
Histology, *n* (%)			.260	–			.272
Adenocarcinoma	1533 (80)	242 (81)			233 (83)	226 (80)	
Squamous	236 (12)	43 (14)			37 (13)	42 (15)	
LCNEC	34 (2)	4 (1)			6 (2)	4 (1)	
Adenosquamous	30 (2)	6 (2)			2 (1)	6 (2)	
Pleomorphic	29 (2)	2 (1)			4 (1)	2 (1)	
Others	50 (2)	3 (1)			0 (0)	2 (1)	
Pathological whole tumour size, cm, median (IQR)	2.5 (1.9-3.2)	2.4 (1.7-3.2)	.336	–	2.5 (1.8-3.2)	2.5 (1.8-3.4)	.595
Lymphatic invasion, *n* (%)	542 (28)	64 (21)	.005	–	76 (27)	61 (22)	.175
Vascular invasion, *n* (%)	740 (39)	95 (32)	.018	–	98 (35)	91 (32)	.532
Pleural invasion, *n* (%)	458 (24)	56 (19)	.103	–	62 (22)	55 (20)	.467
Pulmonary metastasis, *n* (%)	61 (3)	12 (4)	.477	–	11 (4)	11 (4)	1.000
pN factor (eighth), *n* (%)			<.001	–			.011
N0	1600 (84)	269 (90)			241 (86)	251 (89)	
N1	174 (9)	28 (9)			26 (9)	28 (10)	
N2	138 (7)	3 (1)			15 (5)	3 (1)	
Adjuvant therapy, *n* (%)	586 (31)	22 (7)	<.001	–	59 (21)	22 (8)	<.001

Abbreviations: GGO = ground-glass opacity; IQR = Interquartile range; LCNEC = large-cell neuroendocrine carcinoma; MLND = mediastinal lymph-node dissection; SMD = standardized mean difference; SUVmax = maximum standardized uptake value.

### Prognostic impact of MLND

**Figure 1.**
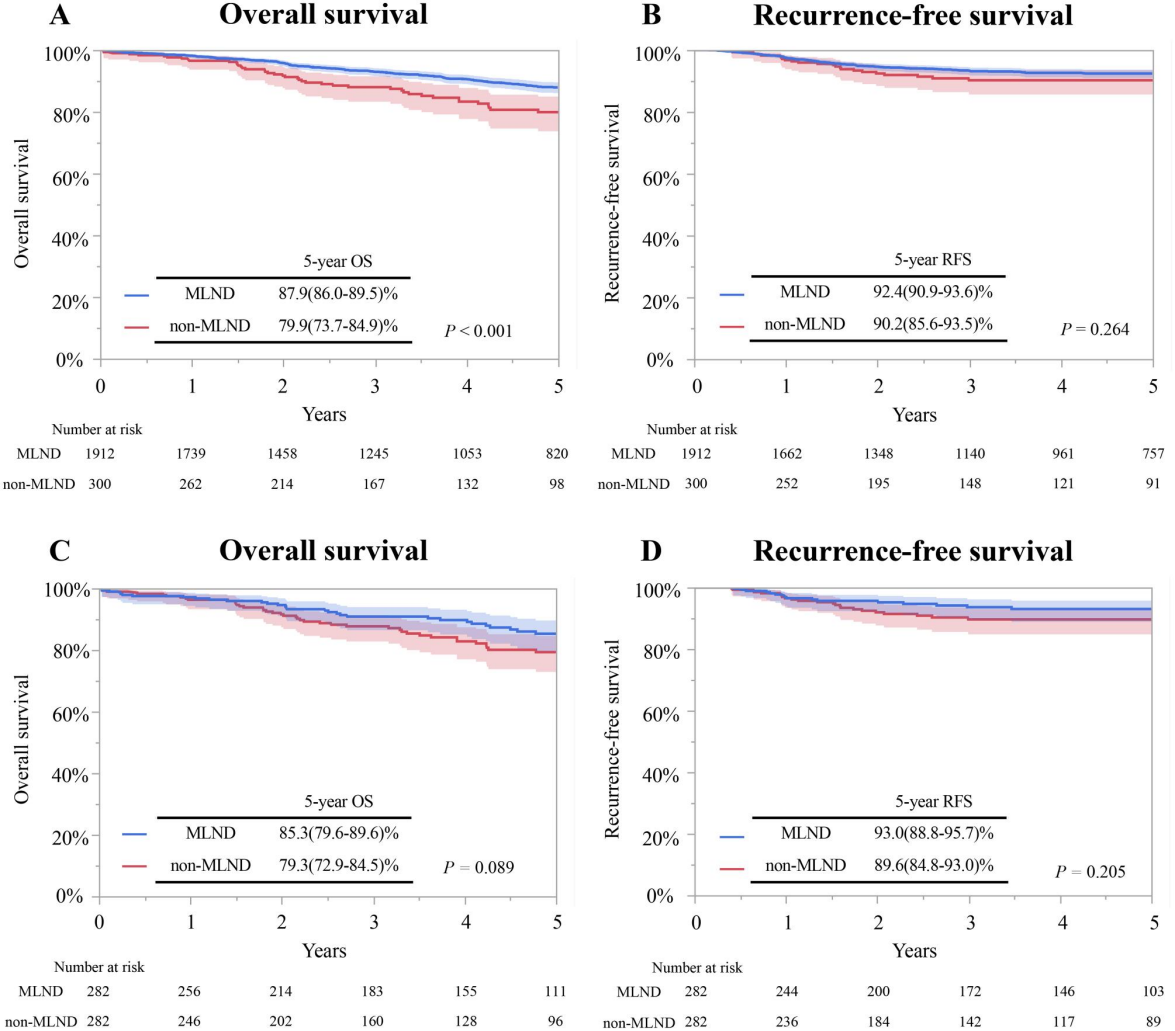
Kaplan-Meier Curves for Overall Survival (OS) and Recurrence-Free Survival (RFS) Before and After Propensity Score Matching (PSM), Comparing Patients With and Without Mediastinal Lymph-Node Dissection (MLND): (A) OS before PSM; (B) RFS before PSM; (C) OS after PSM; and (D) RFS after PSM. The coloured area indicates the 95% CI

**Figure 1** shows the comparison of OS and RFS between the MLND and non-MLND groups. Prior to PSM, with a median follow-up period of 51 (IQR, 24.2-68.0) months, the MLND group demonstrated significantly better OS than the non-MLND group (5-year OS: 87.9% vs 79.9%, *P* < .001). However, no significant difference was found in RFS (5-year RFS: 92.4% vs 90.2%, *P* = .264) between both the groups.

After adjusting for patient characteristics in both the groups using PSM, the median follow-up period was 47.9 (IQR, 22.3-63.5) months. No significant differences were observed in OS (5-year OS: 85.3% vs 79.3%, *P* = .089) or RFS (5-year RFS: 93.0% vs 89.6%, *P* = .205) between both the groups.

Univariable and multivariable analyses for OS and RFS are shown in **Table 2**. In the multivariable analysis considering only preoperative variables, age, sex, SUVmax, and MLND were identified as independent prognostic factors for overall survival (HR for non-MLND: 1.58; 95% CI: 1.14-2.17; *P* = .005). However, when adjuvant therapy was incorporated into the model as a covariate, MLND lost its independent statistical significance for overall survival (HR for non-MLND: 1.37; 95% CI: 0.98-1.94; *P* = .064). Conversely, the administration of adjuvant therapy was a significant independent prognostic factor for overall survival (HR: 0.73; 95% CI: 0.54-0.99; *P* = .043).

**Table 2.** Univariable and Multivariable Cox Proportional Hazards Analysis for OS and RFS

	OS						RFS					
	Univariable analysis		Multivariable analysis		Multivariable analysis		Univariable analysis		Multivariable analysis		Multivariable analysis	
	HR (95% CI)	*P*-value	HR (95% CI)	*P*-value	HR (95% CI)	*P*-value	HR (95% CI)	*P*-value	HR (95% CI)	*P*-value	HR (95% CI)	*P*-value
Age (≥75/<75)	2.02 (1.57-2.59)	<.001	1.84 (1.42-2.39)	<.001	1.77 (1.34-2.33)	<.001	0.83 (0.58-1.18)	.298	1.04 (0.72-1.51)	.827	1.18 (0.80-1.74)	.404
Sex (male/female)	2.54 (1.92-3.34)	<.001	1.83 (1.32-2.53)	<.001	1.85 (1.32-2.59)	<.001	2.07 (1.45-2.96)	<.001	1.64 (1.09-2.48)	.018	1.57 (1.03-2.38)	.035
Pack-year (≥20/<20)	2.13 (1.63-2.78)	<.001	1.23 (0.90-1.67)	.195	1.19 (0.87-1.62)	.275	1.43 (1.02-2.01)	.039	0.81 (0.55-1.19)	.281	0.84 (0.56-1.24)	.374
Solid tumour size (>2 cm/≤2 cm)	2.00 (1.55-2.60)	<.001	1.26 (0.94-1.69)	.129	1.36 (1.00-1.85)	.053	2.88 (1.97-4.19)	<.001	1.77 (1.16-2.70)	.008	1.66 (1.07-2.57)	.022
GGO (absent/present)	2.16 (1.63-2.86)	<.001	1.22 (0.90-1.65)	.208	1.27 (0.93-1.75)	.136	2.22 (1.51-3.27)	<.001	1.19 (0.78-1.80)	.418	1.28 (0.83-1.98)	.256
SUVmax (>4.0/≤4.0)	3.49 (2.65-4.58)	<.001	2.58 (1.89-3.51)	<.001	2.58 (1.88-3.56)	<.001	4.29 (2.89-6.35)	<.001	3.08 (1.99-4.77)	<.001	2.89 (1.85-4.50)	<.001
cT factor (T2/T1)	1.73 (1.32-2.26)	<.001	1.05 (0.78-1.41)	.746	1.08 (0.80-1.46)	.628	2.21 (1.57-3.12)	<.001	1.29 (0.89-1.88)	.184	1.30 (0.89-1.91)	.181
MLND (No/Yes)	1.74 (1.28-2.37)	<.001	1.58 (1.14-2.17)	.005	1.37 (0.98-1.94)	.064	1.29 (0.83-2.01)	.265	1.51 (0.94-2.41)	.086	1.50 (0.92-2.43)	.101
Adjuvant therapy (Yes/No)	0.76 (0.58-1.00)	.049			0.73 (0.54-0.99)	.043	1.55 (1.11-2.17)	.011			1.19 (0.82-1.73)	.369

Abbreviations: GGO = ground-glass opacity; HR = hazard ratio; MLND = mediastinal lymph-node dissection; OS = overall survival; RFS = recurrence-free survival; SUVmax = maximum standardized uptake value.

### Impact of MLND on local control

The recurrence patterns among patients with pN2 in the MLND group are presented in **Table 3**. Among the 1912 patients who underwent MLND, 7.3% (138/1912) were diagnosed with pN2. Of the total MLND cohort, 3.7% (70/1912) experienced recurrence, whereas 3.6% (68/1912) remained recurrence-free. As shown in **Table 4**, patients with pN2 in the MLND group were categorised into the recurrence and non-recurrence groups for comparison. The recurrence group had a significantly higher incidence of intrapulmonary metastases and a higher number of mediastinal lymph-node metastases than the recurrence-free group (*P* = .042 and *P* = .002, respectively), but a lower rate of skip-N2 involvement (*P* = .031).

**Table 3.** Recurrence Patterns in Patients With pN2 From the MLND Group

Recurrence (−)	68 (3.6%)
Recurrence (+)	70 (3.7%)
Locoregional	11 (0.6%)
MLN	7 (0.4%)
Others	1 (0.1%)
MLN + others	3 (0.2%)
Distant	52 (2.7%)
Locoregional + distant	6 (0.3%)
Unknown	1 (0.1%)

Abbreviation: MLND = mediastinal lymph-node dissection.

**Table 4.** Patient Characteristics of pN2 in the MLND Group, Stratified by Recurrence Status

	Recurrence (−) (*n* = 68)	Recurrence (+) (*n* = 70)	*P*-value
Age, years, median (IQR)	67 (59-73)	67 (60-72)	.947
Sex, *n* (%)			.125
Female	31 (46)	23 (33)	
Male	37 (54)	47 (67)	
Pack-year, *n*, median (IQR)	10 (0-41)	30 (0-47)	.170
Side, *n* (%)			.307
Right	42 (62)	49 (70)	
Left	26 (38)	21 (30)	
Clinical whole tumour size, cm, median (IQR)	2.6 (2.1-3.1)	2.8 (2.0-3.3)	.745
Solid tumour size, cm, median (IQR)	2.5 (2.1-2.9)	2.6 (1.8-3.1)	.539
GGO, *n* (%)			.622
Absent	50 (74)	54 (77)	
Present	18 (26)	16 (23)	
SUVmax, median (IQR)	7.5 (3.7-9.7)	7.3 (3.8-10.5)	.332
cT factor (eighth), *n* (%)			.391
T1a	1 (1.5)	2 (2.9)	
T1b	15 (22.0)	20 (28.6)	
T1c	35 (51.5)	26 (37.1)	
T2a	17 (25.0)	22 (31.4)	
Histology, *n* (%)			.062
Adenocarcinoma	62 (91)	52 (74)	
Squamous	2 (3)	9 (13)	
LCNEC	1 (2)	2 (3)	
Adenosquamous	2 (3)	2 (3)	
Pleomorphic	0 (0)	3 (4)	
Others	1 (2)	2 (3)	
Pathological whole tumour size, cm, median (IQR)	2.6 (2.0-3.3)	2.8 (2.1-3.7)	.345
Lymphatic invasion, *n* (%)	48 (71)	57 (81)	.135
Vascular invasion, *n* (%)	49 (72)	57 (81)	.191
Pleural invasion, *n* (%)	27 (40)	34 (49)	.294
Pulmonary metastasis, *n* (%)	3 (4)	10 (14)	.042
Adjuvant therapy, *n* (%)	41 (60)	45 (64)	.629
Lobe			.672
Right upper	18 (27)	25 (36)	
Right middle	3 (4)	5 (7)	
Right lower	21 (31)	19 (27)	
Left upper	16 (24)	12 (17)	
Left lower	10 (15)	9 (13)	
MLND, *n* (%)			.191
ND2a-1	48 (71)	42 (60)	
ND2a-2	20 (29)	28 (40)	
Dissected mediastinal lymph nodes, *n*, median (IQR)	17 (12-23)	17 (12-26)	.203
Mediastinal lymph-node metastases, *n*, median (IQR)	2 (1-4)	4 (2-7)	.002
N2 station, *n* (%)			.063
1	54 (79)	43 (61)	
2	9 (13)	19 (27)	
3	1 (2)	3 (4)	
4	0 (0)	1 (1)	
Unknown	4 (6)	4 (6)	
Skip pN2, *n* (%)	28 (41)	17 (24)	.031

Abbreviations: GGO = ground-glass opacity; MLND = mediastinal lymph-node dissection; SUVmax = maximum standardized uptake value.

A comparison of the recurrence patterns between the MLND and non-MLND groups is presented in **Table 5**. Both the groups had similar recurrence rates of approximately 15% (*P* = .966). Mediastinal lymph-node recurrence was significantly lower in the MLND group (*P* = .017).

**Table 5.** Patterns of Recurrence in Patients With and Without MLND

	MLND (*n* = 1912)	Non-MLND (*n* = 300)	*P*-value
Recurrence			.966
(−)	1627 (85%)	255 (85%)	
(+)	285 (15%)	45 (15%)	
Locoregional	46 (2%)	13 (4%)	.079
MLN	29 (2%)	11 (4%)	.017
Others	10 (0.5%)	1 (0.3%)	
MLN + others	7 (0.4%)	1 (0.3%)	
Distant	201 (11%)	24 (8%)	.217
Locoregional + distant	35 (2%)	4 (1%)	.812
Unknown	3 (0.2%)	4 (1%)	

Abbreviation: MLND = Mediastinal lymph-node dissection.

## DISCUSSION

In this study, we investigated the prognostic impact of MLND in patients with radiologically solid-dominant clinical stage I NSCLC. In the multivariable analysis considering only preoperative variables, MLND was identified as an independent prognostic factor for overall survival (*P* = .005). However, when postoperative adjuvant therapy was incorporated into the model, this statistical significance disappeared (*P* = .064), while adjuvant therapy emerged as a significant prognostic factor (*P* = .043). These statistical results align with the finding that overall survival did not reach statistical significance after propensity score matching (*P* = .089). Consequently, whether MLND itself independently improves overall survival remains uncertain in the present study.

On the other hand, the clinical utility of MLND in achieving superior local control was suggested. In our cohort, MLND significantly reduced mediastinal lymph-node recurrence (*P* = .017). Furthermore, MLND identified occult pN2 metastasis in 7.3% (138/1912) of patients,^14^ and importantly, 3.6% (68/1912) of the total MLND cohort had pN2 disease yet remained completely recurrence-free during the follow-up period. Of these 68 recurrence-free patients, 41 received adjuvant therapy, whereas 27 did not. Notably, the fact that some patients achieved recurrence-free survival even without adjuvant therapy strongly suggests that MLND itself exerted a direct therapeutic effect on preventing local recurrence. This indicates that although MLND may not independently dictate overall survival, it remains useful for local control.

While MLND provides local control, distant metastasis generally accounts for a large proportion of recurrences in NSCLC, indicating the importance of adjuvant therapy for the high-risk groups. In our study, among patients with pN2, the recurrence group exhibited a significantly higher incidence of intrapulmonary metastases and a higher number of mediastinal lymph-node metastases, but a lower rate of skip-N2 involvement, compared to the recurrence-free group. In patients with pN2 demonstrating these features, MLND alone may provide insufficient local control, and adjuvant therapy should be strongly considered to eliminate micrometastatic cancer cells. Recently, adjuvant therapies, including immune checkpoint inhibitors, epidermal growth factor receptor tyrosine kinase inhibitors, and anaplastic lymphoma kinase inhibitors, have demonstrated superior outcomes compared to conventional chemotherapy alone,^15^ further emphasizing the importance of accurate staging through MLND to identify appropriate candidates for these treatments.

The ACOSOG Z0030 trial showed no significant improvement in survival with MLND compared to sampling, reporting unsuspected pN2 metastasis in only 4%.^5^ By focusing on radiologically solid-dominant tumours, we observed a higher rate of occult pN2 metastasis (7.3%). On the other hand, for patients with an extremely low risk of occult metastasis, MLND could potentially be omitted. Specifically, a supplementary analysis of the JCOG0802/WJOG4607L trial reported a pN2 incidence of 5.4% in pure-solid tumours but only 0.5% in part-solid tumours.^16^^,^^17^ Furthermore, previous studies suggest that MLND might be safely omitted in part-solid tumours with an SUVmax < 2.0, or in peripherally located tumours, which carry a lower risk of pN2 than central tumours.^18^^,^^19^ In such cases, the omission of MLND might be justified to reduce surgical invasiveness without compromising oncological outcomes.

Indeed, it is important to acknowledge that MLND carries a potential risk of postoperative complications, such as chylothorax, recurrent laryngeal nerve palsy, and seroma. Concerns regarding such excessive invasiveness may lead some thoracic surgeons to be cautious about performing MLND, particularly in early-stage cases.

This study has several limitations. First, it was a retrospective analysis of prospectively collected data. The decision to perform MLND was based on the surgeon’s discretion, leading to unavoidable selection bias. Second, the database lacked detailed information on potential confounding factors such as pulmonary function, performance status, specific comorbidities, surgical approach, and surgeon’s experience. Third, the MLND status in the database was categorized as non-MLND, selective MLND, or systematic MLND, without specific data on lymph-node sampling. Consequently, the non-MLND group may have included patients who had undergone MLNS. Finally, detailed data on the use of recent novel adjuvant treatments, such as immune checkpoint inhibitors and targeted therapies, were not fully evaluated owing to the long study period. Given their substantial impact on survival, further investigations incorporating these modern systemic therapies are warranted.

In conclusion, while MLND is useful for achieving superior local control and accurate staging in patients with radiologically solid-dominant clinical stage I NSCLC, whether it directly improves overall survival remains uncertain. Future prospective randomized trials are warranted to determine its true survival benefit.

## Data Availability

The data underlying this study are available in this article.

## Abbreviations

CTR: consolidation tumour ratio
FDG-PET/CT: 18-fluoro-2-deoxyglucose positron emission tomography/CT
GGO: ground-glass opacity
HRCT: high-resolution CT
HU: Hounsfield units
IQR: interquartile range
MLND: mediastinal lymph-node dissection
MLNS: mediastinal lymph-node sampling
NSCLC: non-small-cell lung cancer
OS: overall survival
PSM: propensity score matching
RFS: recurrence-free survival
SUVmax: maximum standardized uptake value
